# Insanities of Sane People

**Published:** 1873-01

**Authors:** P. H. Hayes


### INSANITIES OF SANE PEOPLE.

P. H. HAYES, M. D.

> What good, what evil from ourselves proceed,
> How doth the subtle principle within,
> Inspire with health, or mine with strange decay,
> The passive body. [ARMSTRONG.

Many years ago a Paris correspondent of the English *Court Journal* gave an account of the departure of Eugenie, then Empress of France, *incognita* for Scotland. The reason for this journey was the sad state of mental depression into which the Empress had fallen, on account of the then recent death of her sister, the Duchess of Alba. Medical prescriptions from the most distinguished Parisian physicians failed in her case, and nothing but the exercises of religion could give her any calmness or consolation. In these exercises she would spend the greater part of every night in her favorite chapel at St. Cloud.

The delusion of the Empress was that she herself would soon die of the same lingering and painful disease as her sister, and characteristic of this state of mind she began to experience all the symptons of her sister's disease, as suggested by her imagination, unbalanced by reason and tyrannized over by fear. This painful mental condition is known in France as *l'imagination frappee—imagination-stricken.* The Duchess of Hamilton, passing through Paris, repaired to St. Cloud to pay her respects to her illustrious relative, when perceiving the very serious nature of her mental and bodily suffering, hastened to get the Emperor's consent, and it is said arranged everything in an hour; and it is added that the haste and agitation of her departure, with the thousand objects of beauty and interest she saw in passing through the streets of Paris, she had not seen in so long, began to produce that violent *distraction*—diversion from herself to outward things—which, even before she left France, had broken the spell and rendered her recovery certain.

I stepped out into the bright moonlight to take a walk with my friend, Professor J——, who was weak in one knee. Becoming deeply engaged in conversation, we continued our walk back and forth in front of the house, for some time, when he suddenly exclaimed "What a *walk* I am taking!" and standing for a moment to compute the probable distance he had actually traveled, he said, "if I had seen the length of my walk before me at once, it would have been impossible. My brains would have been all down in my knee!" I examined the knee with great care, and found it perfectly free from any disease, and I discovered that he had through fear, unconsciously, mentally withheld its natural supply of nerve power, and being made to see this, he soon afterward quite recovered the strength of the joint.

Curiously, enough, fear seems to bring on symptoms of disease in the part to which the attention is directed. Thus in the case of the Empress, she is said to have had, in a degree, the same symptoms as her sister, though she did not truly have any disease at all. And I now remember that in a Doctor's family—with whom I am well acquainted—the servant was sent to a drawer in the office to get soda to put in the biscuit for tea. When the biscuits appeared they were, as the housewife said, "heavy as lead." After the guests had tasted the biscuit all around, it occurred to some one to inquire whether "Biddy" had not made a mistake and put in something else, they knew not what, poison! perhaps, from the wrong drawer, into the biscuit. Instantly, this fear so struck the imagination, that eating was at an end. Some began to vomit the little food they had taken, and were unable to leave the sofa. In the midst of the scene the Doctor returned and settled it, that Biddy had indeed made a mistake, but she had used only the perfectly harmless *sugar of milk* instead of the soda. Of course the reaction from such violent fear was a most hearty laugh all around.

These illustrations have in them volumes of meaning, as going to show how dispositions of mind do constantly influence bodily health, and specially to show that many diseases which appear at first sight to be only bodily in their origin and character, are found on more deep and careful inquiry, to be largely, if not entirely, mental.

It must not be forgotten that the imagination, when stricken by grief, fear, hate, or shame has the power to produce actual bodily sufferings. This physicians recognize, but often only in a superficial way, and while they talk of the "kinks" and "crotchets" of their patients, they forget that their pains are real, and that therefore to tell them their disease is imaginary, is simply to ask your patient to believe you against the evidence of his senses, and to withhold sympathy and turn such a patient off with a laugh, is often the very refinement of cruelty.

Among all the insanities of sane people, there are none more distressing than those in which fear gives the blow and causes the suffering. I am acquainted with a gentleman who has for years made himself and family restless and wretched through fear of poverty, while it is well known to all who know him that he has most ample means for any contingency that could arise.

A young man who came under my care a few years ago, had studied theories of diet until he became afraid to eat almost any article of food that could be named. Under this fear he left off one after another all the articles of food in common use, until he would eat only some coarse, weak and watery food twice a day, and lest this should be too much for him he would have it weighed, to be sure that he did not overload his stomach. This process caused a slow, but sure and painless starvation.

Nothing hurts the character of children more or is a greater cruelty to them than the habitual appeal to fear in governing them. In this way the better part of all true and noble child-nature is repressed, the child is driven to deceit and concealment, and the virtues which give beauty and frankness and robustness to character, are continually, and often hopelessly dwarfed. Not alone herein do parents need to be on their guard, but also that their young children be not told stories which greatly excite the imagination by fears. Not unfrequently books contain these fear-exciting stories, and we know at this moment two boys, almost young men, who cannot be persuaded to retire to their bed-chambers alone, under any circumstances. If left for a moment alone, they seem to become terror-stricken, and to suffer great agony of mind from fear. I know a man who is more than forty years of age, who, when he is in ill health, suffers from those vague, ideal fears so keenly, that he has told me that a *real* fear, one he could see and somehow measure, was a relief, a real tonic to his mind. Let not the stout-hearted ridicule these fears. It is less than a year since that a father in a neighboring city determined his little son should be cured of his foolish fear of going to bed in the dark alone, and so he shut him up in his room, and would not relent a word, for all his terrible cries to be let out, or have his brother come to sleep with him. After a while all noise ceased in the little fellow's room, and the father opened the door, hoping to find his son asleep and the cure complete. But can you imagine his dismay and grief, when, crouched in one corner of the room, he sees his boy, bereft of reason, a hopeless idiot!

There is a fact about the insanities of mankind which it may do much good to point out. If we go into the hospitals, where those who are so imagination-stricken as not to be allowed a life of freedom are confined, we shall find curiously enough that Mr. B——, who believes he is made of glass, knows that Mrs. K——, who thinks she is Queen Elizabeth, is a fool and laughs at her folly, and Mrs. K—— believes with all her soul that she is truly Queen Elizabeth. She walks about with an air of grand dignity, flourishing her sceptre, but what a fool is Mr. B——, who thinks he is made of *glass!* The late Dr. James Johnson, of London, relates that when visiting the wards of an asylum at Florence, he was escorted through the building by a Barrister on one side and a Priest on the other, both confined there, and victims of harmless delusions. They were men of talents and learning. They described in the most affecting terms the delusions of the poor lunatics confined there, and not a word escaped either of them indicating a stricken imagination or a disordered mind, until they came to a man who believed himself to be Jesus Christ. The Barrister then seized the Doctor's arm, and casting a look of contempt upon the Priest, said: "I thank my stars I am free from those superstitions which make the vulgar mind the victim of priestly tyranny. I worship the sun, the moon, the stars." At this moment the Priest drew the Doctor forcibly aside. "You now see, sir," said he, "that the unhappy and lost wretch who deals out his impious and atheistic creed, is as complete a maniac as you will find in this whole establishment. *I*, sir, am the only individual in this vast asylum who is in his perfect senses!" The Priest soon betrayed his own peculiar delusion. So it is in human life, among all who walk the earth, each has some great or small delusion; but each sees not his own, only his neighbors, and like the Priest, is apt to say, in manner, if not in words, "I am the only man in all the world who is in his perfect senses." Young has it—

All men think all men mortal but themselves.

